# Current and Future Land Use around a Nationwide Protected Area Network

**DOI:** 10.1371/journal.pone.0055737

**Published:** 2013-01-31

**Authors:** Christopher M. Hamilton, Sebastian Martinuzzi, Andrew J. Plantinga, Volker C. Radeloff, David J. Lewis, Wayne E. Thogmartin, Patricia J. Heglund, Anna M. Pidgeon

**Affiliations:** 1 Department of Forest and Wildlife Ecology, University of Wisconsin, Madison, Wisconsin, United States of America; 2 Department of Agricultural and Resource Economics, Oregon State University, Corvallis, Oregon, United States of America; 3 Economics Department, University of Puget Sound, Tacoma, Washington, United States of America; 4 Upper Midwest Environmental Sciences Center, United States Geological Survey, La Crosse, Wisconsin, United States of America; 5 Upper Midwest Environmental Sciences Center, United States Fish and Wildlife Service, La Crosse, Wisconsin, United States of America; University of Western Australia, Australia

## Abstract

Land-use change around protected areas can reduce their effective size and limit their ability to conserve biodiversity because land-use change alters ecological processes and the ability of organisms to move freely among protected areas. The goal of our analysis was to inform conservation planning efforts for a nationwide network of protected lands by predicting future land use change. We evaluated the relative effect of three economic policy scenarios on land use surrounding the U.S. Fish and Wildlife Service's National Wildlife Refuges. We predicted changes for three land-use classes (forest/range, crop/pasture, and urban) by 2051. Our results showed an increase in forest/range lands (by 1.9% to 4.7% depending on the scenario), a decrease in crop/pasture between 15.2% and 23.1%, and a substantial increase in urban land use between 28.5% and 57.0%. The magnitude of land-use change differed strongly among different USFWS administrative regions, with the most change in the Upper Midwestern US (approximately 30%), and the Southeastern and Northeastern US (25%), and the rest of the U.S. between 15 and 20%. Among our scenarios, changes in land use were similar, with the exception of our “restricted-urban-growth” scenario, which resulted in noticeably different rates of change. This demonstrates that it will likely be difficult to influence land-use change patterns with national policies and that understanding regional land-use dynamics is critical for effective management and planning of protected lands throughout the U.S.

## Introduction

Humans have modified over 83% of the Earth's land surface due to land-use [1]. Changes in land-use practices, and more specifically, conversion of land from more natural conditions to less natural conditions is one of the main threats to biological diversity [2], [3]. The alteration of land cover and subsequent appropriation of the Earth's resources has major effects on climate, water, and biodiversity, and these in turn affect management of fish and wildlife resources [3]–[5]. Intensive land use, which we defined as areas where natural cover has been converted into pasture, crop, or urban use, affects biodiversity through both habitat loss and fragmentation [2], [6], [7], altering community composition [8], [9], limiting species ranges [10], restricting animal dispersal and migration [6], [11], [12], and facilitating invasion by non-native species [13], [14].

Land use also threatens the value and effectiveness of protected areas as a conservation tool [15], [16]. Conservation efforts rely heavily on protected areas to provide refugia that safeguard biodiversity [17]. However, protected areas are linked to their surroundings by ecological flows (i.e., of energy, organisms, material) and processes and do not exist in isolation [18]. The effectiveness of protected areas for conserving biodiversity is therefore influenced by the surrounding landscape, which is often altered by land use such as agriculture and settlements that typically eliminate, degrade, and fragment habitats [15], [19], [20]. On the other hand, abandonment of agricultural lands can provide opportunities for habitats to be restored, with a potential positive effect on populations of native species.

Given that surrounding land use affects the function of protected areas, it is important to understand drivers of change, as well as threats and opportunities that those changes may pose to the maintenance of biodiversity [21]–[23]. Furthermore, understanding future land use around protected areas is crucial to effectively mitigating potential effects of climate change given that many climate change adaptation strategies call for establishment of corridors to allow for species migration as suitable habitat and environmental conditions shift location [19], [22]. Future land-use change is a vital consideration when investing limited conservation funds [24], [25].

Understanding land use and land-use change around protected areas at different spatial scales (i.e., buffers) is important to have a better understanding of human pressures on protected areas [22], [26]. It is also important because species relate to the landscape in different ways. Species differ in their home range size requirements, movement and dispersal capabilities, and perception of the environment. Therefore, it is important to understand land-use change at different scales that correspond to the range of scales at which species relate to landscapes [23], [27]–[29].

Land-use change very broadly follows a trajectory from natural land cover to frontier clearing, subsistence agriculture, and ending in intensive land use where the majority of land has been converted for agricultural and urban use. Different regions of the world are at different points along this trajectory and the time required to pass through the different stages varies widely, with some regions remaining in the frontier and subsistence stage [30]. The United States, however, has progressed through the stages, beginning with agricultural production, followed by growth of population centers, and finally urbanization and has established regional land use patterns [31], [32]. Because these patterns are predictable to some degree, future land use can be simulated. Regional patterns may vary considerably and changes in land use are affected by regional economic, demographic, and ecological forces which, when modeled, allow us to fine-tune simulations of future land use [33]. Past changes in land use provides information that can be exploited to quantify the likely effects of different economic policies and scenarios on future land-use patterns [34]. In the United States, past land-use trends suggest that land use is likely to continue to intensify rapidly, with urban use growing faster than other land-use classes [16]. This means that the United States protected area network may be at risk from the effects of land-use intensification surrounding protected areas.

In the U.S., the only federally owned network of protected areas designed primarily for the protection of fish, wildlife, and plants is the U.S. Fish and Wildlife Service's (USFWS) National Wildlife Refuge System (NWRS). Other Federal lands (e.g., those owned by U.S. Forest Service, Bureau of Land Management, or National Park Service) are managed for multiple purposes. The main goal of the NWRS is to maintain the biological integrity of the refuge system [35] yet this goal may be compromised by intensifying land use in the surroundings of the refuges [36]. Furthermore, given concerns that climate change will likely exacerbate current land-use effects on wildlife and the refuge system, it is of great interest to the USFWS to assess future land-use change as a major step in determining what climate change adaptation measures are indicated [19]. Predictive models of future land-use change can help managers explore plausible outcomes of different policy or economic scenarios and are thus an important tool for maintaining biological integrity. Future scenarios generated from such models can provide land managers with a better understanding of the full range of potential futures, and of the possible effects of alternate futures on biodiversity and other ecological resources [37]–[40]. Managers may be able to use the scenario outcomes to identify important pre-emptive actions for lands outside protected area boundaries which, if managed appropriately can be important for maintenance of biodiversity [21], [41].

Our goal here was thus to evaluate current and future (yr. 2051) land use around the National Wildlife Refuges in the contiguous 48 United States. Specifically, we asked four questions:

- First, what is the current land use in the areas surrounding the National Wildlife Refuges?
- Second, what will the likely differences in surrounding land use be in 2051 under different economic and policy scenarios?
- Third, how do future conditions under these scenarios vary among regions or extent of analysis?
- Finally, what are the primary threats and opportunities to the NWRS in each of the seven USFWS administrative regions in the contiguous 48 states?

## Methods

### 2.1 Study Area

We focused on USFWS National Wildlife Refuges in the conterminous United States (hereafter the refuges). We excluded NWRS lands that were not directly managed by USFWS (i.e., cooperatively managed lands) or specifically designated as refuges in the USFWS Cadastral database (http://www.fws.gov/GIS/data/CadastralDB/), which resulted in 461 refuges for our analyses.

We modeled land-use change at different scales around the refuges to assess whether the scale of analysis affected the land-use change trends. Changes were analyzed within 5, 25, and 75 km of each refuge. Some areas fell within the analyzed distances for more than one refuge (e.g., some areas were within 75 km of two or more refuges). These areas were counted more than once when calculating values for each of the 461 refuges, but were counted only once when calculating values for the refuge system as a whole. We chose these buffer distances because they approximate movement distances during the three main annual habitat use stages of the mallard (*Anas platyrhynchos*), a species that benefits a great deal from NWRS management. This choice reflects our effort to link our analyses to something biologically meaningful to managers in most of the NWRS, but we do not mean to imply that all refuges are managed for mallards only. The 5-km distance encompasses most brood movements [42], 25 km approximates the daily foraging movements of overwintering ducks [43], and 75 km is representative of the post-breeding/pre-migration movements [44]. The maximum distance also equates to the distance within which many private land habitat restoration projects supported by the USFWS have been completed. The USFWS has implemented a private lands habitat restoration program called “Partners for Fish and Wildlife” for 25 years, which names as one of its priorities projects that benefit the NWRS and includes over 1,000,000 acres of restored wetlands as of 2010 (for further information see http://www.fws.gov/partners/).

### 2.2 Analyses

We quantified future land-use change around the refuges using an econometric land-use model [34], [45]. The model used observed land-use changes from the National Resources Inventory (NRI) between 1992 and 1997, measures of soil productivity, and county-level net economic returns to estimate land-use transition probabilities for urban, forest, range, crop, and pasture lands from 2001to 2051. Sets of transition probabilities were estimated separately for each county and soil quality class, and account for feedback effects of land-use changes on commodity prices and, thus, the net economic return to each use. The transition probabilities were combined with the National Land Cover Dataset (NLCD, 2001) to develop fine-scale projections of land use. The range category in the NRI was matched to the grassland/herbaceous and shrubland categories in the NLCD. An important advantage of using an econometric model as a basis for predicting change around refuges is that it permits us to evaluate the effect of different economic policy scenarios on land-use change. For instance, a subsidy for afforestation will alter the net return for forested land and, thus, the transition probabilities among the different land uses.

We considered three different scenarios of future land-use change in our analyses. The scenarios were designed to represent ambitious but potentially plausible policies that have already been implemented in some areas (e.g., some metropolitan areas have urban growth boundaries comparable to the restricted-urban-growth scenario). The first was “business as usual”, which applied the transition probabilities of the base model. These transition probabilities reflected the land-use change that occurred between 1992 and 1997, the time period on which the econometric model parameters were estimated. The second scenario, referred to as “preserve-natural-habitats,” was a conservation policy that levied a $100/acre tax on land that leaves forest or range use (i.e., a tax on land leaving natural vegetation). In this case, we assumed forest and range lands to be in a natural state. The third scenario, “restricted-urban-growth,” was a policy scenario in which urban growth was restricted to already urbanized counties (specifically, counties that are part of a metropolitan statistical area). The land-use model assumed no transition out of urban use since there were no such transitions observed in the NRI data [46]. In addition, the model did not include a transition into or from barrens or open water, as these areas normally remain in the same use. Also, wetlands were assumed to not transition to other uses since these areas also tend to remain in the same use, being protected by state and Federal regulations. Only private lands were subject to change in our analyses, i.e., land use in protected areas remained constant for the period of study. An assumption of the model was that responses by private landowners to changing economic conditions in the 1990s provide a basis for predicting responses to future economic conditions.

We evaluated the land use changes for our three scenarios in three major land uses: natural (NLCD classes 41, 42, 43, 52, and 71), agriculture (NLCD classes 81 and 82), and urban (NLCD classes 21–24). We conducted these analyses for the refuge system as a whole as well as for the 7 different administrative regions of the USFWS in the contiguous United States.

We looked at change in three ways. The first was the absolute change in area that occurred for a particular land use. The second was the change in the percentage of the buffer area represented by each land use. The final value was the rate of change for each land use over the 50 year span of our analysis.

Our projections of land-use accounted for endogenous feedbacks on the prices of market outputs from land, a key improvement over the Radeloff et al. [33] land-use projections. The feedbacks resulted from land-use induced shifts in the supply of key outputs produced from land: agricultural products, timber, and housing. For example, if the amount of cropland declined, then the resulting decrease in the supply of crops induced an increase in crop prices. Any change in the price of an output from a particular land-use thus altered the net returns to that use, and affected future land-use change. We adopted the approach originally developed by Lubowski et al. [44] to incorporate endogenous price feedbacks for the key land-uses in our analysis: crops, pasture, range, forest, and urban uses.

## Results

### 3.1 Current use

**Figure 1:**
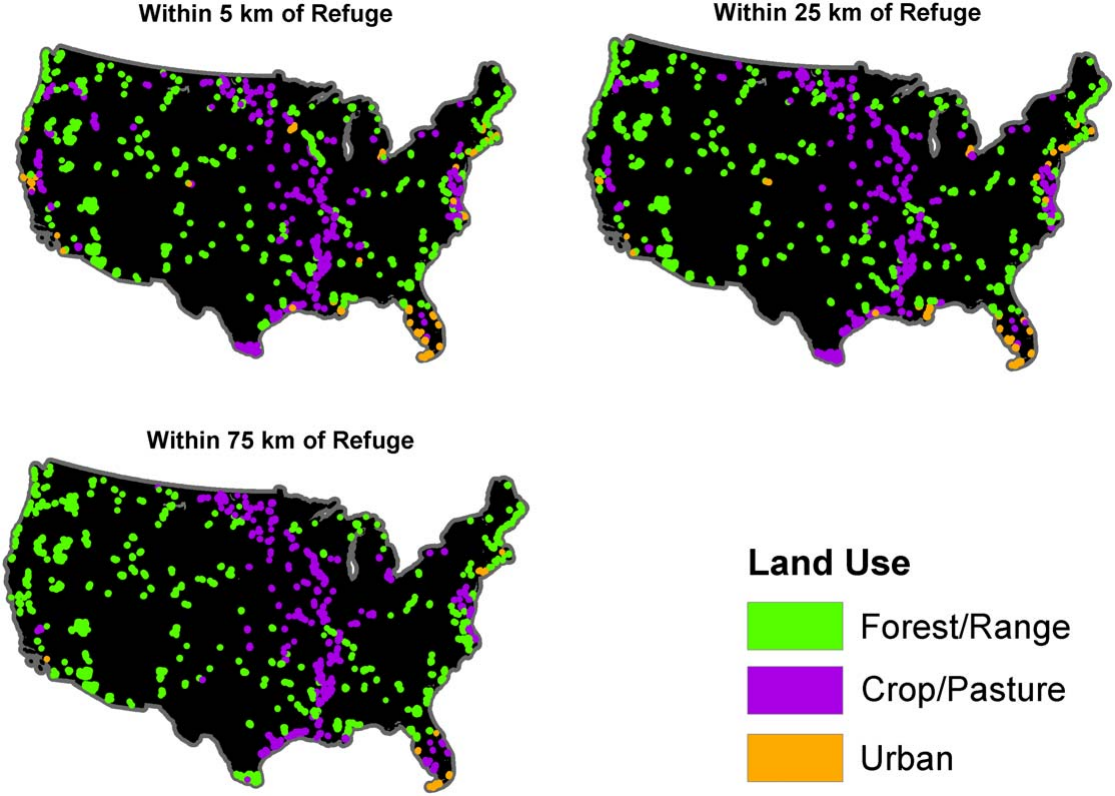
Current dominant land use around each National Wildlife Refuge in the contiguous 48 states.

**Figure 2:**
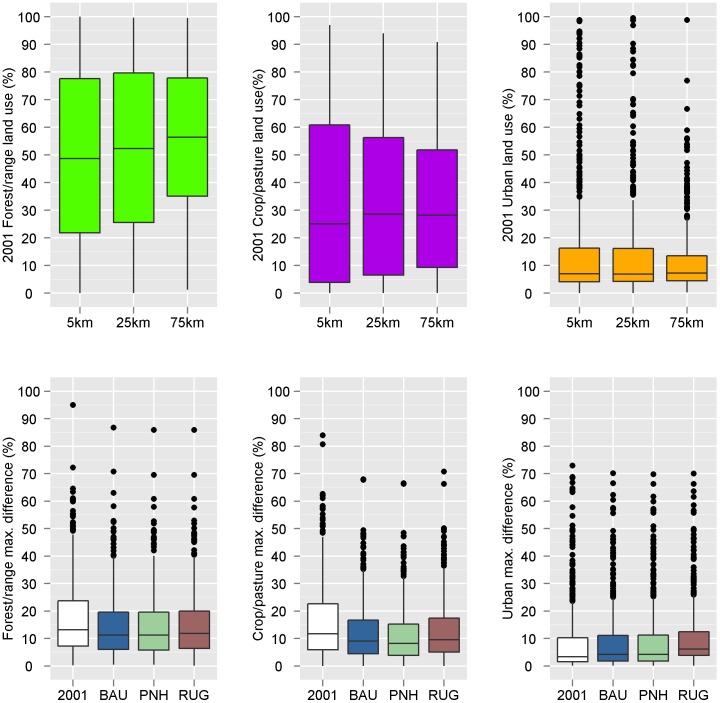
Box plots showing the variability in each land use across the scales of analysis for different land-use classes (row 1), and the maximum difference among scales in proportion of land in each use around the National Wildlife Refuges (row 2). Plots in row 2 compare conditions in 2001 (in white) with those in 2051 for each scenario (BAU = business-as-usual, PNH = preserve-natural-habitats, RUG = restricted-urban-growth).

Forest/range was the dominant land use surrounding the refuges in 2001, encompassing 57.4% (58.3 million ha) of the area within 25 km of all refuges, with crop/pasture following at 24.6% (34.2 million ha) and urban use the lowest at 6.5% (8.9 million ha). The remaining 24.4% of land use consisted of open water, wetlands, barrens, and non-natural woody cover such as orchards or vineyards. The dominant land use around NWRS lands varied both regionally and by the scale of analysis (Table 1, Figure 1). For example, along the coasts, urban use dominated within 5 km of some refuges but at 25 and 75 km forest/range or crop/pasture were the dominant land cover types (Figure 1). The proportion of refuges for which forest/range was the dominant use increased with increasing scale of analysis, while the proportion of crop/pasture remained relatively stable and dominance by urban land use decreased with increasing scale of analysis. For many individual refuges, the dominant land use changed across the scales of analysis (Table 1, Figure 1). The median proportion of land around the refuges in urban use and crop/pasture remained relatively stable across scales (between 25% and 30%), while the median proportion of land in forest/range use increased slightly with increasing scale from 49% to 56% (Figure 2). Increasing the scale of analysis smoothed the variability in the range of predicted urban use values, where the median value remained approximately 7%, but the range of values decreased with increasing scale (Figure 2). However, the scale of analysis had minimal effect on the proportion of refuges dominated by a particular use and on the median value for most land uses (Table 1, Figure 1, Figure 2). Because this was true for starting conditions as well as conditions in 2051 for each scenario, we report further results only at the 25-km scale.

**Table 1:** Percent of National Wildlife Refuges dominated by different land-use categories, at three different scales (buffer distances) and the proportion of each use category on all private lands nationwide.

	Distance around Refuge (km)			
Land use	5	25	75	National percentage
Forest/Range	53.2	56.9	63.6	57.8
Crop/Pasture	36.8	34.8	33.0	34.6
Urban	10.0	8.3	3.5	7.6

### 3.2 NWRS overall

In each of the three future scenarios, forest/range and urban land use increased while crop/pasture use declined. In all scenarios, the rate of urban growth, which was between 28.5% and 57.0%, far outstripped the rate of change of either forest/range or crop/pasture. The scenarios affected the particular land uses differently (Table 2). Forest/range land use experienced similar increases under both the preserve-natural-habitats and the restricted-urban-growth scenarios but a smaller increase under the business-as-usual scenario. Crop/pasture loss was highest under the preserve-natural-habitats scenario (Table 2). Most notably, urban land use grew at the lowest rate under the restricted-urban-growth scenario, exhibiting a nearly 50% reduction in the rate of increase, relative to other scenarios. In terms of area, crop/pasture losses were the greatest change followed by urban land use increases and, finally, forest/range increases (Table 2). Those changes, however, were not evenly distributed among the refuges.

**Table 2:** Land area (million ha) and percent areain different land uses within 25 km of the National Wildlife Refuges in 2001 and 2051 under each scenario.

			Business-as-Usual		Preserve-natural-habitats		Restricted-urban-growth	
Land Use	Area 2001	% Area 2001	Area 2051	% Change	Area 2051	% Change	Area 2051	% Change
Forest/Range	58.3	57.4	59.4	1.9	61.0	4.7	60.9	4.5
Crop/Pasture	34.2	24.6	28.0	−17.0	25.9	−23.1	29.0	−15.2
Urban	8.9	6.5	13.7	52.2	14.2	57.0	11.6	28.5

### 3.3 Regional patterns

**Figure 3:**
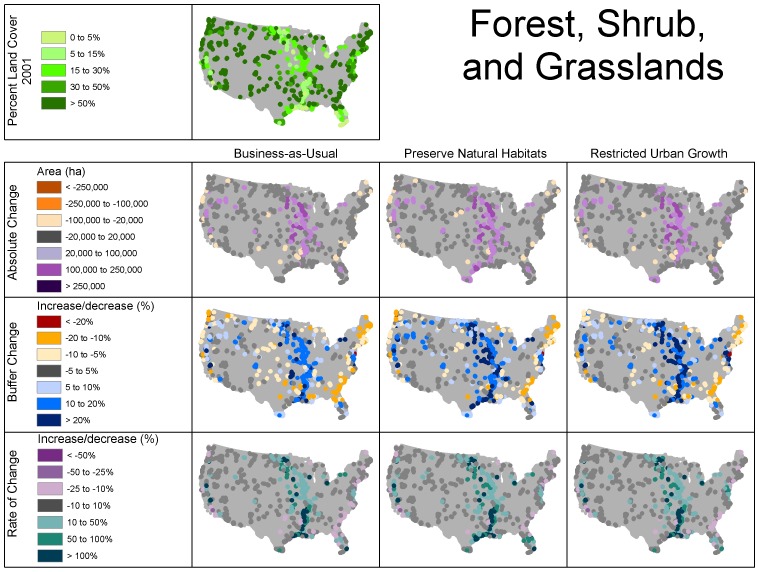
Initial land use and change in forest/range land use within 25 km of refuges under the different scenarios. Absolute change = change in area (ha), buffer change = percent of buffer that changes use, and rate of change = percent growth.

**Figure 4:**
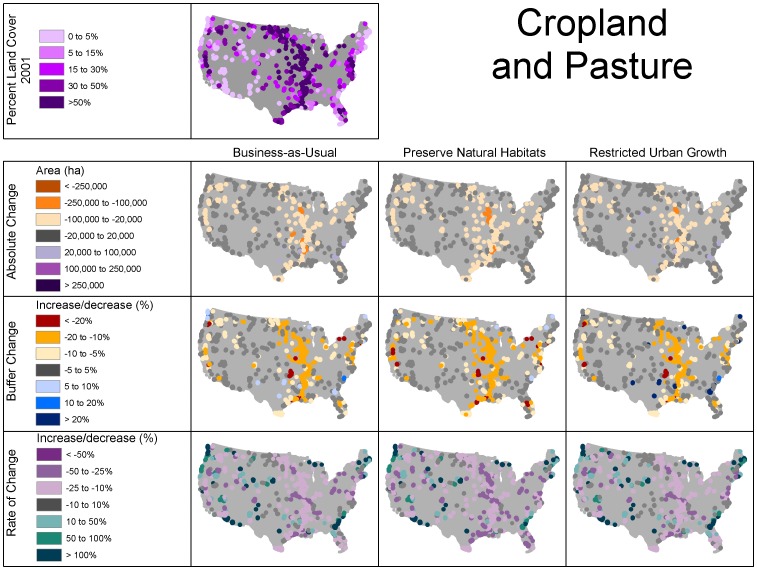
Initial land use and change in crop/pasture land use under the different scenarios.

**Figure 5:**
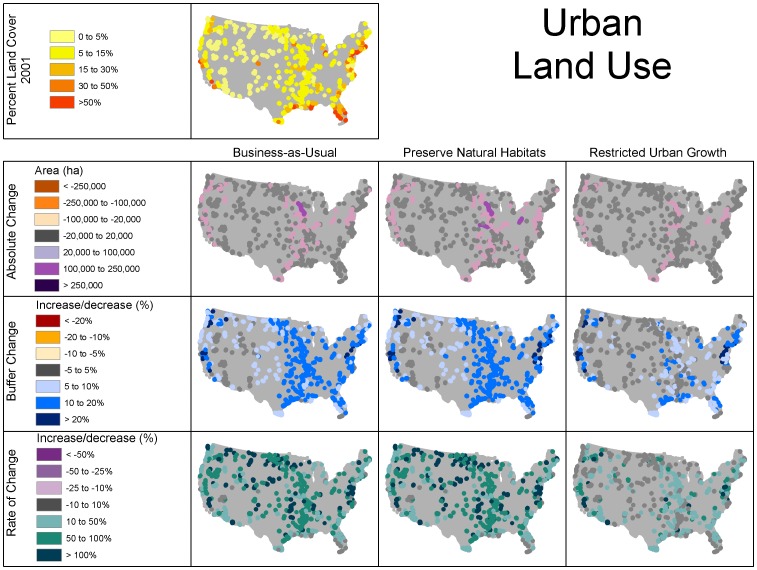
Initial land use and change in urban land use under the different scenarios.

Clusters of refuges surrounded primarily by forest/range in 2001 were located mainly in the western half of the U.S., as well as near the Appalachian mountains in both the southeast and northeast, whereas refuges surrounded primarily by crop/pasture in 2001 were concentrated in the Midwest and along the Mississippi River (Figures 1, 3, and 4). The classification scheme we used for percent land use somewhat obscured urban land use, because it is relatively less prevalent, but several clusters were apparent in the Western U.S., as well as on the Atlantic and Gulf coasts of Florida and in the mid-Atlantic states (Figure 5).

The increase in forest/range land was greatest in terms of absolute change, proportion of surrounding area, and rate of change in the Upper Midwest, while losses of forest/range were less frequent with no regional pattern (Figure 3). This increase in forest/range use came at the expense of crop/pasture (Table 2). Forest/range land change was most prevalent in the Midwest (Figure 3). The increase in forest/range change strongly coincided with decreases of crop/pasture (Figure 4). Urban land use is unique in that it is persistent and did not decrease under any scenario (Figure 5). Total area and percent of buffer area increased in urban land use also coincided with areas of crop/pasture loss. However, while areas of high growth rates for urban use included the areas of crop/pasture loss, the areas with the highest rates of growth were much more widely distributed owing to the fact that rates of change can be relatively higher in areas with low urban land use (Figure 5).

**Figure 6:**
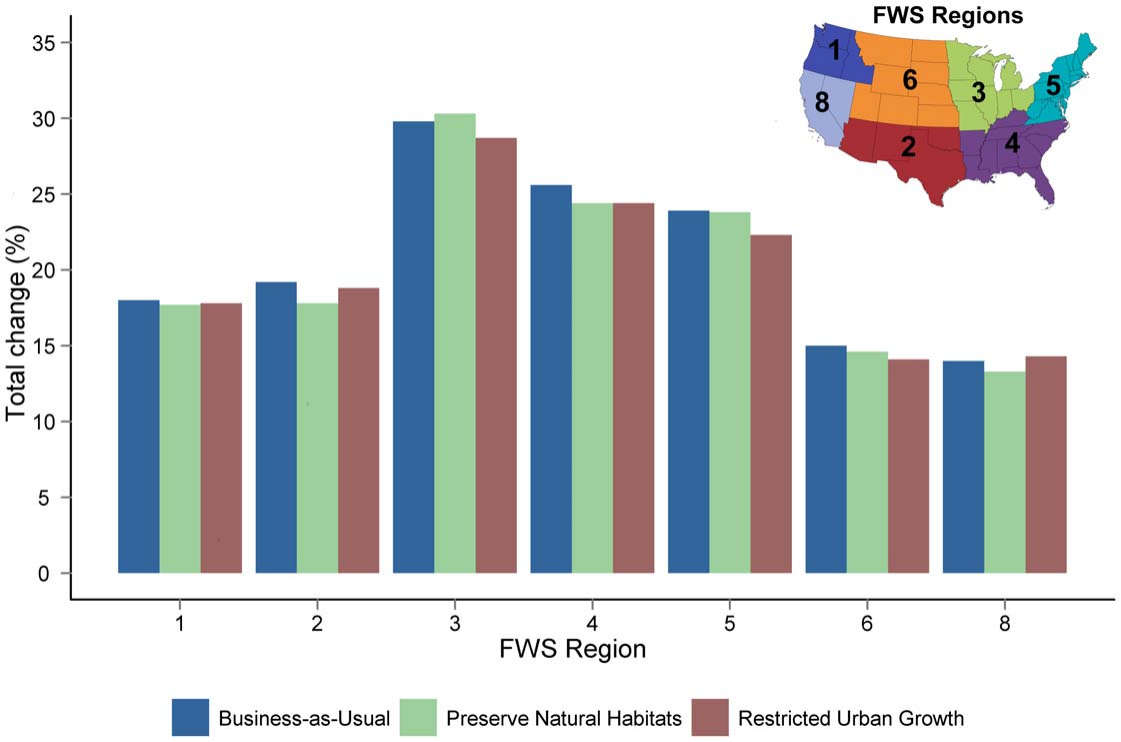
Regional predictions for the percentage of area within 25 km of refuges that will change under the different scenarios. Inset shows FWS administrative regions.

We also found pronounced differences among FWS administrative regions in percent of total area that changed land use under the different scenarios. Our analyses indicated that region 3 (Upper Midwest region of the United States) stands out as the most dynamic, while changes were relatively similar across the other regions (Figure 6). This pattern occurred in all scenarios. The business-as-usual scenario resulted in the most dynamic change for all but region 3. The land-use response to restricted-urban-growth and preserve-natural-habitats scenarios varied among the regions.

**Figure 7:**
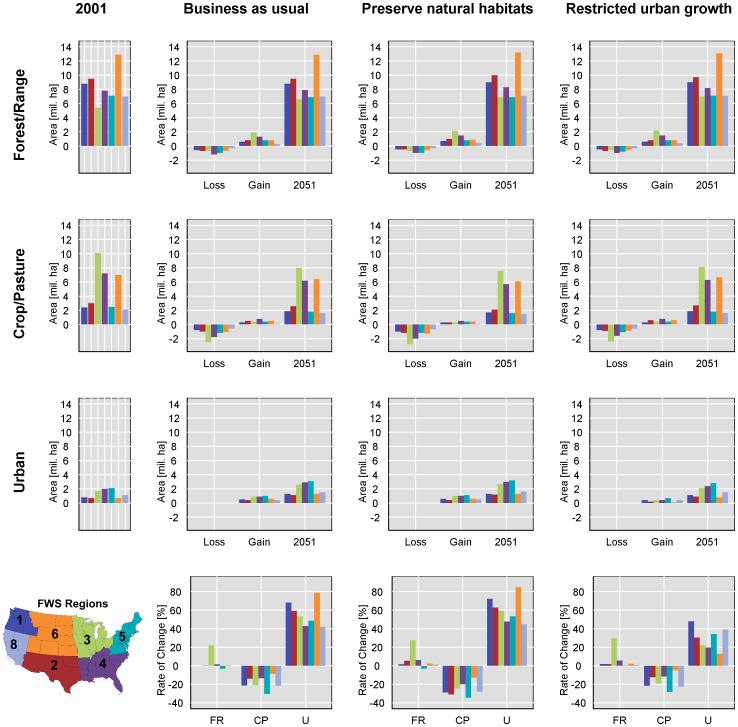
Change in area in different land uses and rates of change among the different scenarios for the FWS administrative regions. FR is forest/range, CP is crop/pasture, and U is urban land use.

The most notable gains in forest/range use area occurred in FWS region 3. Substantial forest/range loss, as a proportion of the surrounding area within 25 km, occurred within FWS regions 4 and 5 under all scenarios (Figure 4, Figure 7). Similarly, the biggest loss in crop/pasture area occurred in region 3, but loss was also notable in the Central Valley of California, and loss of this land use occurred in all regions. Urban growth by absolute area was remarkably similar within regions and when comparing the business-as-usual and restricted-urban-growth scenarios. However, the restricted-urban-growth scenario substantially reduced urban land use increases in area.

The rate of change in forest/range area was slightly higher under both the preserve-natural-habitats and restricted-urban-growth scenarios, but the most striking difference was by geographic region. Region 3 exhibited a much greater rate of increase than the other regions (Figure 7). Most noteworthy was the difference in growth rates among land uses. Urban use increased at a much higher rate than the other uses, nearly doubling in several regions under the business-as-usual and restricted-urban-growth scenarios. Crop/pasture loss rates were very similar under the business-as-usual and restricted-urban-growth scenarios and highest under the preserve-natural-habitats scenario.

## Discussion

We predicted substantial future land-use change in the areas surrounding the National Wildlife Refuge System in the United States by 2051 and identified important threats and opportunities at a regional scale. These threats are relevant to regional managers, and to budget allocations at the federal level, because they show that most of the changes are regional in nature and any attempt to respond to anticipated threats will require a regional response through both policy establishment and budget allocation. While the models indicated that gains and losses will occur in forest/range and crop/pasture, the largest areal changes will be crop/pasture land-use loss. Urban land use was predicted to experience the highest rate of change, though the total urban areal change is likely to be smaller than changes in forest/range or agricultural cover. These trends varied among the scenarios that we analyzed, with forest/range largely gaining at the expense of crop/pasture, simply because land never leaves urban use. The only way to gain forest/range is thus through abandonment of crop/pasture. The most significant difference from the business-as-usual scenario occurred in the case of the restricted-urban-growth scenario, where reductions in housing growth rates were substantial and resulted in as much forest/range increase as under a scenario designed to preserve natural habitats. Our findings were similar to forecasts of crop/pasture loss and forest and urban gain trends forecast by other researchers [34], [47].

Our regional analyses indicated that forest/range land use will likely decrease in the Northeastern U.S., which also agreed with previous findings [48]. The forest/range will likely be lost to urbanization. Finally, the high rate of urban growth was in agreement with other studies reporting high rates of housing growth around protected areas [16], [20], [49].

Given that many studies indicate intensive land use encroaching on protected areas [16], [50], [51], including the NWRS [19], [36], it was a surprise to find that much of the land surrounding the NWRS is currently in forest/range land use. While we considered these to be relatively “natural”, this assumption may not always hold. Low levels of intensively used land can impact the quality of wildlife habitat in nearby areas [13], [52], [53].

We were also surprised to find that the different scenarios had a notable effect on land-use change around the NWRS given that previous analyses using this model found minimal change among scenarios for nationwide projections [34]. However, the land-use projection method that we used was updated to incorporate endogenous price feedbacks and lower rates of urbanization, and we simulated different scenarios that resulted in more notable differences than those reported by Radeloff et al. [33]. In our analyses, the most notable contrast to business-as-usual occurred under the restricted-urban-growth scenario where urban land use growth was restricted to metropolitan counties. It is interesting that restricting urban growth resulted in similar increases in forest/range land use as the preserve natural habitat scenario. This suggests that the effect of unrestricted sprawl on habitat loss is so strong that a policy which restricts urban development, while unlikely to be widely implemented, could deliver similar conservation results as the direct preservation of natural habitat.

The regional variation that we found in terms of the percent of area around refuges that is likely to change concurred with other studies that indicated that protected areas are often protected more by their location than by policies [15]. The other major result was that urban land use will likely be a continuing management challenge for the NWRS. This result is in agreement with other studies predicting continued urban expansion around protected areas and into areas of natural vegetation, thus exacerbating biodiversity loss [54]. Urban growth is a land use that can be difficult to address with incentive-based policies simply because the economic returns are much higher than for other land uses [34]. However, state-adopted urban containment policies that employ strict growth boundaries have been shown to significantly reduce sprawl and preserve open space, with Portland, Oregon being an example of successful containment of urban sprawl [55], [56].

The similarity of the proportion of land currently in each use across the different scales of analysis for the NWRS was surprising to us. However, while our analyses did not suggest significant differences across the U.S., there were substantial regional and contextual differences in dominant land use as well as land-use change. Land-use change was forecasted to be most dynamic in the Midwestern region, followed by the Northern Great Plains region. While previous studies indicated stability in crop use in these regions [32], recent studies have found loss of crop land throughout here [47]. The changes are the result of a transition from crop/pasture use to either urban or forest/range use [34], [47]. Our prediction of reduced land area in crop/pasture may not reflect increasing demands for food and biofuels production though, which are rapidly increasing and can only be met through either agricultural intensification or expansion [57]. In fact, other studies predict increases in crop as well as urban land use worldwide as well as in the Upper Midwest, an area where our models predict decreases in crop land use [58], [59].

Perhaps the most striking result of our analyses was the rate of urbanization. Even with strong policies to restrict urban growth, many regions (1, 2, 5, and 8) are likely to experience high rates of growth in urban land use. While the predicted rates of urban growth under the restricted-urban-growth scenario were substantially reduced when compared with the other scenarios, those rates of growth were still high, matched only by rates of afforestation in the Upper Midwest.

It is worth noting that many of the National Wildlife Refuges are found along the coasts. These areas have a great deal of open water and wetlands, which we excluded from our analyses. Some of our results can give the misleading impression that those refuges are surrounded by urban land use when, in fact, much of the neighboring area is open water or wetland. We suggest though that exclusion of those areas was appropriate given the model goals of evaluating the effect of policies on terrestrial land use.

### 4.1 Model limitations

As any model, our land use model is an abstraction of reality, and our results have thus to be interpreted in the context of model assumptions and limitations. First of all, it is important to state that our “business-as-usual” scenario reflects “business-as-of-the-1990s”. The model was parameterized using data from land-use change between 1992 and 1997. The economic climate in the U.S. in the early 2010s is much different, with higher crop prices and much slower housing growth. However, our scenarios simply reflect adjustment of the model outputs through changing the net economic return for a particular land use. The main assumption in this approach is that human behavior has not changed and is focused on maximizing economic return from land use. The best use for the model is thus the comparison among scenarios and we treat conditions from the 1990s as a scenario that simply reflects what was occurring during a particular period of time.

Second, our model did not calculate confidence intervals for the predictions. Our predicted land use changes were made for the entire conterminous U.S., not just a sample of locations. As such, *any* difference in land use among scenarios is statistically significant, but that does not mean of course that any difference would be significant for management or policy considerations, or that our predictions were free of uncertainty. There are two sources of error that affect our predictions. The first is that the NRI data, upon which the econometric model was based, represents a sample, and as such the land use transition have confidence intervals. The second is that the land cover data (NLCD) has classification error. Given the nature of these types of error, confidence intervals could potentially be derived by simulating many instances of each scenario and sampling the input data from a distribution. Practically, this was not feasible though for two reasons. First, the computational resources required to simulate many instances of each scenarios were far beyond what was available to us. Second, errors in the land cover data are not spatially random [60], but the NLCD provides only aggregate accuracy values, and that makes it impossible to simulate realistic land cover maps based on the available accuracy data.

Third, we recognize that climate change is likely to affect future changes in land use to some extent. The same projection model we used was also used to investigate the effects of future changes in climate on land-use changes in the U.S. by incorporating into the model trends in population, income, agricultural prices, and forestry and crop yields as projected in two IPCC scenarios [61]. Relative to a reference scenario with no changes in climate, climate change had only a small effect on the area of land in broad land-use categories (crops, pasture, forest, urban, and range). For example, across the conterminous U.S., the areas of land in crops and forest were 2.1% and 1.3% lower under the IPCC A1 scenario compared to the reference case, though somewhat larger differences are found in some regions. Within a land-use category, climate change could affect landowner choices such as the types of crops and trees to plant [62], but the findings suggest no large shifts in broad land-use categories [61]. Nevertheless, climate change, or other changes such as new technologies, could result in novel conditions in the future that our model, which was based on past land use trends, could not capture.

## Conclusions

This was, to our knowledge, the first analysis of its kind providing spatially explicit predictions of economic policy scenarios for a nationwide system of protected areas. We have presented these results to managers in the USFWS from the field level to the national level and they have been well-received, most likely because we included managers from the USFWS in the formulation of our proposal, conducting our analyses and compiling results. In addition, our results have been provided in spreadsheet form to the managers in USFWS, stressing though in the accompanying documentation that the results should be used with caution when attempting to establish local policy since any national land use simulation will have limitations when applied at much finer scales. These data include estimates of land-use change for each of the NWRs under each scenario and is easily interpreted for direct use by managers, who possess local knowledge of surrounding land-use pressures, to assess changes in land use that may threaten the lands they manage. Armed with this knowledge, managers can encourage development of policies that address regional and local land-use change concerns. It should be noted that the model was used was developed specifically for the 48 contiguous United States, which has already reached the “intensive” land-use stage. This type of model would not be appropriate for areas which are still in the frontier stage of land-use change [30]. However, we see great possibilities for comparable models to provide similar insights outside the 48 contiguous United States in regions that are in the intensive land-use stage.

Our results indicated that managers will be faced with both opportunities and challenges. Some regions experienced an increase in forest/range lands under all scenarios, which we considered to be more compatible with habitat and biodiversity conservation. In light of the anticipated need for improved habitat connectivity and corridors to mitigate the effects of climate change [19], this is good news for conservation efforts in those regions. Forest regrowth provides valuable habitat and, consequently, conservation opportunities [63], [64]. In fact, our findings are somewhat reassuring for conservationists given that land-use change is currently the main driver of biodiversity loss, and will remain so at least until the mid-century [65], [66]. However, the general increase in forest cover is accompanied by the likely large increase in urban land use under all scenarios. Even with potential improvements in habitat connectivity, the overall implications for biodiversity are not clear. The biodiversity threats posed by urban development are likely to reduce, or even overwhelm, any gains through increased habitat area and connectivity. In fact, managers need to prepare for the challenges of working with increasing numbers of neighbors in a landscape of forest with an understory of houses. Further research on the quality of natural habitat with an intermix of houses and on identifying thresholds of housing density beyond which land ceases to support biodiversity will be important to assist managers of the future [52], [53].Additionally, our results indicated that there are serious limitations to “one-size-fits-all” approaches to policy development and implementation. Our finding that regional differences were as strong as differences among policy scenarios has important implications for management of large networks of lands. This should serve as a caution to policy makers that regional context is critical for land management and that national policies need to be flexible enough to incorporate these differences. At a minimum, managers and policy makers need to assess threats and opportunities to inform optimal allocation of resources. Given these regional differences, it will be critical for managers to work with local and regional governments and partners to minimize the impact of future land use change on their lands. Finally, we suggest that it would be useful to identify buffer zones around refuges within which managers could focus additional conservation efforts through work with neighboring land owners. Managers could work with partners to delineate buffer zones appropriate to conserving those species and their habitats on which a refuge's management focuses, with particular consideration given to the threats likely to occur within that zone.

## References

[1] Sanderson EW, Jaiteh M, Levy MA, Redford KH, Wannebo AV, et al (2002) The human footprint and the last of the wild. Bioscience 52: 891–904.

[2] Fischer J, Lindenmayer DB (2007) Landscape modification and habitat fragmentation: A synthesis. Global Ecology and Biogeography 16: 265–280.

[3] Vitousek PM, Mooney HA (1997) Human domination of earth's ecosystems. Science 277: 494.

[4] Theobald DM, Goetz SJ, Norman JB, Jantz P (2009) Watersheds at risk to increased impervious surface cover in the conterminous United States. Journal of Hydrologic Engineering 14: 362–368.

[5] Williams JW, Jackson ST (2007) Novel climates, no-analog communities, and ecological surprises. Frontiers in Ecology and the Environment 5: 475–482.

[6] Damschen EI, Haddad NM, Orrock JL, Tewksbury JJ, Levey DJ (2006) Corridors increase plant species richness at large scales. Science 313: 1284–1286. 16946070 10.1126/science.1130098

[7] Fahrig L (2003) Effects of habitat fragmentation on biodiversity. Annual Review of Ecology Evolution and Systematics 34: 487–515.

[8] Attum O, Lee YM, Roe JH, Kingsbury BA (2008) Wetland complexes and upland-wetland linkages: Landscape effects on the distribution of rare and common wetland reptiles. Journal of Zoology 275: 245–251.

[9] Pidgeon AM, Radeloff VC, Flather CH, Lepczyk CA, Clayton MK, et al (2007) Associations of forest bird species richness with housing and landscape patterns across the USA. Ecological Applications 17: 1989–2010. 17974337 10.1890/06-1489.1

[10] Schulte LA, Pidgeon AM, Mladenoff DJ (2005) One hundred fifty years of change in forest bird breeding habitat: Estimates of species distributions. Conservation Biology 19: 1944–1956.

[11] Eigenbrod F, Hecnar SJ, Fahrig L (2008) The relative effects of road traffic and forest cover on anuran populations. Biological Conservation 141: 35–46.

[12] Fahrig L (2007) Non-optimal animal movement in human-altered landscapes. Functional Ecology 21: 1003–1015.

[13] Gavier-Pizarro GI, Radeloff VC, Stewart SI, Huebner CD, Keuler NS (2010) Rural housing is related to plant invasions in forests of southern Wisconsin, USA. Landscape Ecology 25: 1505–1518.

[14] Predick KI, Turner MG (2008) Landscape configuration and flood frequency influence invasive shrubs in floodplain forests of the Wisconsin river (USA). Journal of Ecology 96: 91–102.

[15] Joppa LN, Loarie SR, Pimm SL (2008) On the protection of “protected areas”. Proceedings of the National Academy of Sciences of the United States of America 105: 6673–6678. 18451028 10.1073/pnas.0802471105 PMC2365567

[16] Radeloff VC, Stewart SI, Hawbaker TJ, Gimmi U, Pidgeon AM, et al (2010) Housing growth in and near united states protected areas limits their conservation value. Proceedings of the National Academy of Sciences 107: 940–945. 10.1073/pnas.0911131107 PMC2818924 20080780

[17] Gaston KJ, Jackson SF, Cantú-Salazar L, Cruz-Piñón G (2008) The ecological performance of protected areas. Annual Review of Ecology, Evolution, and Systematics 39: 93–113.

[18] Hansen AJ, DeFries R (2007) Ecological mechanisms linking protected areas to surrounding lands. Ecological Applications 17: 974–988. 17555212 10.1890/05-1098

[19] Griffith B, Scott JM, Adamcik R, Ashe D, Czech B, et al (2009) Climate change adaptation for the US national wildlife refuge system. Environmental Management 44: 1043–1052. 19548023 10.1007/s00267-009-9323-7

[20] Wade AA, Theobald DM (2010) Residential development encroachment on US protected areas. Conservation Biology 24: 151–161. 19624528 10.1111/j.1523-1739.2009.01296.x

[21] Franklin JF, Lindenmayer DB (2009) Importance of matrix habitats in maintaining biological diversity. Proceedings of the National Academy of Sciences of the United States of America 106: 349–350. 19129497 10.1073/pnas.0812016105 PMC2626705

[22] Heller NE, Zavaleta ES (2009) Biodiversity management in the face of climate change: A review of 22 years of recommendations. Biological Conservation 142: 14–32.

[23] Wiens JA (2009) Landscape ecology as a foundation for sustainable conservation. Landscape Ecology 24: 1053–1065.

[24] Merenlender AM, Newburn D, Reed SE, Rissman AR (2009) The importance of incorporating threat for efficient targeting and evaluation of conservation investments. Conservation Letters 2: 240–241.

[25] Smith B, Burton I, Klein RJT, WANG IJ (2000) An anatomy of adaptation to climate change and variability. Climate Change 45: 223.

[26] Burgman MA, Lindenmayer DB, Elith J (2005) Managing landscapes for conservation under uncertainty. Ecology 86: 2007–2017.

[27] D'Eon R, Glenn SM, Parfitt I, Fortin MJ (2002) Landscape connectivity as a function of scale and organism vagility in a real forested landscape. Conservation Ecology 6: 10.

[28] Olden JD, Schooley RL, Monroe JB, Poff NL (2004) Context-dependent perceptual ranges and their relevance to animal movements in landscapes. Journal of Animal Ecology 73: 1190–1194.

[29] Sutherland G, Harestad A, Price K, Lertzman K (2000) Scaling of natal dispersal distances in terrestrial birds and mammals. Conservation Ecology 4: 16.

[30] Foley JA, DeFries R, Asner GP, Barford C, Bonan G, et al (2005) Global consequences of land use. Science 309: 570–574. 16040698 10.1126/science.1111772

[31] Huston MA (2005) The three phases of land-use change: Implications for biodiversity. Ecological Applications 15: 1864–1878.

[32] Brown DG, Johnson KM, Loveland TR, Theobald DM (2005) Rural land-use trends in the conterminous united states, 1950–2000. Ecological Applications 15: 1851–1863.

[33] Sohl TL, Loveland TR, Sleeter BM, Sayler KL, Barnes CA (2010) Addressing foundational elements of regional land-use change forecasting. Landscape Ecology 25: 233–247.

[34] Radeloff VC, Nelson E, Plantinga AJ, Lewis DJ, Helmers D, et al (2012) Economic-based projections of future land use in the conterminous united states under alternative policy scenarios. Ecological Applications 22: 1036–1049. 22645830 10.1890/11-0306.1

[35] Meretsky VJ, Fischman RL, Karr JR, Ashe DM, Scott JM, et al (2006) New directions in conservation for the national wildlife refuge system. Bioscience 56: 135–143.

[36] Scott JM, Loveland T, Gergely K, Strittholt J, Staus N (2004) National wildlife refuge system: Ecological context and integrity. Natural Resources Journal 44: 1041–1066.

[37] Coreau A, Pinay G, Thompson JD, Cheptou P, Mermet L (2009) The rise of research on futures in ecology: Rebalancing scenarios and predictions. Ecology Letters 12: 1277–1286. 19874385 10.1111/j.1461-0248.2009.01392.x

[38] Fleishman E, Blockstein DE, Hall JA, Mascia MB, Rudd MA, et al (2011) Top 40 priorities for science to inform US conservation and management policy. Bioscience 61: 290–300.

[39] Gude PH, Hansen AJ, Jones DA (2007) Biodiversity consequences of alternative future land use scenarios in Greater Yellowstone. Ecological Applications 17: 1004–1018. 17555214 10.1890/05-1108

[40] White D, Minotti PG, Barczak MJ, Sifneos JC, Freemark KE, et al (1997) Assessing risks to biodiversity from future landscape change. Conservation Biology 11: 349–360.

[41] Cox RL, Underwood EC (2011) The importance of conserving biodiversity outside of protected areas in mediterranean ecosystems. Plos One 6: e14508. 21249126 10.1371/journal.pone.0014508 PMC3017544

[42] Mauser DM, Jarvis RL, Gilmer DS (1994) Movements and habitat use of mallard broods in northeastern California. Journal of Wildlife Management 58: 88–94.

[43] Davis BE, Afton AD (2010) Movement distances and habitat switching by female mallards wintering in the Lower Mississippi alluvial valley. Waterbirds 33: 349–356.

[44] Gaidet N, Cappelle J, Takekawa JY, Prosser DJ, Iverson SA, et al (2010) Potential spread of highly pathogenic avian influenza H5N1 by wildfowl: Dispersal ranges and rates determined from large-scale satellite telemetry. Journal of Applied Ecology 47: 1147–1157.

[45] Lubowski R, Plantinga A, Stavins R (2006) Land-use change and carbon sinks: Econometric estimation of the carbon sequestration supply function. Journal of Environmental Economics and Management 51: 135–152.

[46] Nusser S, Goebel J (1997) The national resources inventory: A long-term multi-resource monitoring programme. Environmental and Ecological Statistics 4: 181–204.

[47] Pijanowski BC, Robinson KD (2011) Rates and patterns of land use change in the upper great lakes states, USA: A framework for spatial temporal analysis. Landscape and Urban Planning 102: 102–116.

[48] Drummond MA, Loveland TR (2010) Land-use pressure and a transition to forest-cover loss in the eastern united states. Bioscience 60: 286–298.

[49] Radeloff VC, Hammer RB, Stewart SI, Fried JS, Holcomb SS, et al (2005) The wildland-urban interface in the united states. Ecological Applications 15: 799–805.

[50] Hansen AJ, DeFries R (2007) Land use change around nature reserves: Implications for sustaining biodiversity. Ecological Applications 17: 972–973. 17555211 10.1890/05-1112

[51] Leinwand IIF, Theobald DM, Mitchell J, Knight RL (2010) Landscape dynamics at the public-private interface: A case study in Colorado. Landscape and Urban Planning 97: 182–193.

[52] Gagne SA, Fahrig L (2010) The trade-off between housing density and sprawl area: Minimizing impacts to carabid beetles (coleoptera: Carabidae). Ecology and Society 15: 12.

[53] Gagne SA, Fahrig L (2010) The trade-off between housing density and sprawl area: Minimising impacts to forest breeding birds. Basic and Applied Ecology 11: 723–733.

[54] Seto KC, Fragkias M, Gueneralp B, Reilly MK (2011) A meta-analysis of global urban land expansion. Plos One 6: e23777. 21876770 10.1371/journal.pone.0023777 PMC3158103

[55] Woo M, Guldmann J (2011) Impacts of urban containment policies on the spatial structure of US metropolitan areas. Urban Studies 48: 3511–3536.

[56] Nelson A, Moore T (1996) Assessing growth management policy implementation - case study of the united states' leading growth management state. Land use Policy 13: 241–259.

[57] Tilman D, Balzer C, Hill J, Befort BL (2011) Global food demand and the sustainable intensification of agriculture. Proceedings of the National Academy of Sciences 108: 20260–20264. 10.1073/pnas.1116437108 PMC3250154 22106295

[58] Nelson E, Sander H, Hawthorne P, Conte M, Ennaanay D, et al (2010) Projecting global land-use change and its effect on ecosystem service provision and biodiversity with simple models. Plos One 5: e14327. 21179509 10.1371/journal.pone.0014327 PMC3002265

[59] Sohl TL, Sleeter BM, Sayler KL, Bouchard MA, Reker RR, et al (2012) Spatially explicit land-use and land-cover scenarios for the great plains of the united states. Agriculture, Ecosystems & Environment 153: 1–15.

[60] Comber A, Fisher P, Brunsdon C, Khmag A (2012) Spatial analysis of remote sensing image classification accuracy. Remote Sensing of Environment 127: 237–246.

[61] Haim D, Alig RJ, Plantinga AJ, Sohngen B (2011) Climate change and future land use in the united states: An economic approach. Climate Change Economics 2.

[62] Sohngen B, Mendelsohn R (1998) Valuing the impact of large-scale ecological change in a market: The effect of climate change on US timber. American Economic Review 88: 686–710.

[63] Bowen ME, McAlpine CA, House APN, Smith GC (2007) Regrowth forests on abandoned agricultural land: A review of their habitat values for recovering forest fauna RID C-8155-2009 RID A-3907-2010. Biological Conservation 140: 273–296.

[64] Bowen ME, McAlpine CA, Seabrook LM, House APN, Smith GC (2009) The age and amount of regrowth forest in fragmented brigalow landscapes are both important for woodland dependent birds RID D-2992–2009. Biological Conservation 142: 3051–3059.

[65] van Vuuren DP, Sala OE, Pereira HM (2006) The future of vascular plant diversity under four global scenarios. Ecology and Society 11: 25.

[66] Sala OE, Chapin FS, Armesto JJ, Berlow E, Bloomfield J, et al (2000) Biodiversity - global biodiversity scenarios for the year 2100. Science 287. 10.1126/science.287.5459.1770 10710299

